# Under‐Identification of Malnutrition Within an Inpatient Hospital Setting

**DOI:** 10.1111/jhn.70338

**Published:** 2026-08-26

**Authors:** Jacqui Bailey, Jolie Baird, Olivia Dullard, Sophie Kane, Caroline J. Tuck, Katherine J. Desneves

**Affiliations:** ^1^ Nutrition and Dietetics Department, Division of Allied Health Austin Health Heidelberg VIC Australia; ^2^ Department of Allied Health, School of Health Sciences Swinburne University Hawthorn VIC Australia

**Keywords:** guideline, malnutrition, prevalence, referral, risk, screening, systems

## Abstract

**Introduction:**

Malnutrition contributes to significant health and economic burden. A 2022 Malnutrition Point Prevalence Survey (PPS) at Austin Health, Melbourne, reported a 40% prevalence rate, consistent with international studies. However, despite robust screening and referral processes, 28% of at‐risk or malnourished patients were not receiving dietetic care on the PPS day. This study aimed to evaluate the rate and timing of electronic malnutrition screening tool (eMST) completion in adult inpatients against local hospital guidelines, compare the accuracy of nursing completed eMST with dietitian completed MST and identify common sources of error in screening completion and potential contributors for those patients who were identified as at risk of or with malnutrition and not receiving dietetic care.

**Methods:**

A retrospective case series of dietitian referrals, weights, time of eMST completion and MST results between admission and the PPS day were assessed for timeliness against hospital guidelines and accuracy based on available weight history, dietitian completed MST and nutrition impact symptoms documented in the electronic medical record (EMR).

**Results:**

On the day of the PPS 48 patients at‐risk of (*n* = 19) or malnourished (SGA B *n* = 27, SGA C *n* = 2) were not receiving dietetic care. Of these, 60% (*n* = 29) missed dietetic care due to inaccurate MST completion. No weight loss (*n* = 20) was incorrectly reported despite patient reports or EMR data indicating otherwise. 35% of patients (*n* = 17) were weighed within 24 h of admission as per hospital guideline. 65% of MSTs completed during the morning nursing shift were inaccurate, compared with 48% during the afternoon and 36% during the night shift.

**Conclusion:**

This study examining the alignment between real‐world screening practices and institutional guidelines for patients at risk of malnutrition identified clear gaps in the timely and accurate completion of MST screening and weight documentation. A multitude of factors likely influence this including nursing staff capacity, flawed design of EMR systems, and suboptimal protocol sequencing. Future research applying systems‐level thinking is required.

## Practitioner Points

- Systems‐level thinking is required to improve the accuracy of malnutrition screening.
- Identification of malnutrition and malnutrition risk could be improved by optimising hospital protocol sequencing to ensure weight is obtained prior to completing malnutrition screening and increasing the visibility of weight history within the electronic medical record.

## 1 Introduction

Malnutrition is a condition characterised by a deficiency, excess or imbalance of essential nutrients necessary for proper bodily function [1]. The prevalence of malnutrition in Australia is estimated to be 30%–60% in acute care [2], 30%–50% in rehabilitation [3, 4], and 30%–70% in residential aged care settings [5]. However, exact prevalence rates are not known due to differences in malnutrition screening, assessment, and diagnostic methods [6, 7]. Malnutrition is associated with negative health outcomes including increased risk of infections, co‐morbidities, mortality, length of hospital admissions and readmissions, and a decline in functional status and quality of life [8]. Within the Australian health care setting, malnutrition is 1 of 16 complications that cause financial penalties for hospitals [9]. The cost of malnutrition in public hospitals is estimated to be $9 billion per annum [5].

Malnutrition screening is a core expectation of Australian healthcare systems under the National Safety and Quality Health Service (NSQHS) Standards which mandates systematic identification, assessment and management of nutritional risk to prevent harm [10]. At an organisational level, these requirements are operationalised through local hospital guidelines that specify screening tools, roles, timeframes and referral pathways. However, the effectiveness of these processes depends on the accuracy and consistency of screening, not just completion.

The Malnutrition Screening Tool (MST) developed in Australian hospital populations is widely used due to its simplicity and demonstrated validity [11, 12]. However, evidence suggests that its performance may be reduced in routine clinical practice. For example, when nurses completed MST without standardised malnutrition risk screening training ~36% of patients were identified at risk of malnutrition compared with ~54% identified by dietitian completed MSTs, there was only fair agreement between raters (*k* = 0.297) and moderate correlation (*rs* = 0.465) [13]. Another study comparing MST completed by patients and dietitians found high overall agreement (*k* = 092), however there was 4%–6% disagreement with repeat administration by patients. Although MST avoids calculation errors seen with more complex tools, such as the Malnutrition Universal Screening Tool (MUST) [14, 15, 16, 17], its accuracy remains influenced by human factors, including training, patient recall and clinician interpretation, with implications for NSQHS compliance. Inaccurate screening may result in missed identification and delayed dietetic referral, limiting the effectiveness of nutrition care despite apparent guideline adherence.

Despite widespread use of the MST, there is a limited evidence base examining its accuracy in routine clinical practice. Existing research has largely focused on validation and reliability under controlled conditions [11, 12, 18], with few studies quantifying real‐world errors in MST completion or their clinical impact. In contrast to the extensive audit literature for MUST [14, 15, 16, 17], there is a lack of studies comparing documented MST scores with independently verified assessments. NSQHS standards and local guidelines must be supported by system‐level enablers to ensure screening processes are not only completed, but accurate, reproducible, and actionable. Despite the recognition of these issues, there remains limited evidence examining the accuracy of screening tool completion in routine practice, particularly in relation to adherence to local policy and national standards. Therefore, the aims of this study in patients who were identified as at risk of or with malnutrition and not receiving dietetic care were to:

i. Evaluate the rate and timing of electronic MST (eMST) completion in adult inpatients against local hospital guidelines.
ii. Compare the accuracy of nursing completed eMST with dietitian completed MST.
iii. Identify common sources of error in screening completion and potential contributors.

By addressing these aims, this study seeks to inform targeted system‐level interventions to improve malnutrition risk identification and delivery of nutrition care in alignment with NSQHS standards.

## 2 Materials and Methods

The study protocol for this retrospective case series was approved by the Austin Health Human Research Ethics Committee (HREC/103922/Austin‐2023) and complied with STROBE reporting guidelines [19]. This retrospective case series was an exploratory subset analysis of patients deemed at risk of malnutrition or diagnosed with malnutrition and not receiving dietetic care on the day of an internal malnutrition point prevalence survey (PPS).

Hospital guidelines specify that patients are weighed within 24 h of admission and every 7 days thereafter, or in accordance with clinical need. Hospital guidelines also outline the expectation that nurses screen patients for malnutrition risk using the eMST within 8 h of admission, every 7 days, and with any change in clinical condition and/or physical location. At Austin Health, a specific dietitian referral logic from the eMST has been implemented to ensure that patients who are in most need of dietetic assessment receive an automatic referral. The eMST comprises two questions, one relating to unintentional weight loss and the other relating to loss of appetite [11]. For the weight‐loss item, responses are scored as follows: no (0), unsure [2], or yes, with 1–5 kg scored as 1, 6–10 kg as 2, 11–15 kg as 3, and > 15 kg as 4. The appetite item is scored as no (0) or yes [11]. A referral is triggered when specific combinations of weight loss and appetite risk are present. Automatic referrals occur when a patient reports unintentional weight loss > 6 kg regardless of appetite change, or when any amount of unintentional weight loss is reported in combination with decreased appetite. In practice, when the nurses complete the eMST shortly after admission, a measured weight is often not yet available, meaning the nurse must rely on the patient's self‐reported response to the weight loss question. When patients do not reach the threshold to generate an automatic eMST referral, a manual dietitian referral may still be placed by nurses, medical or allied health staff if other clinical concerns exist, such as suspected chronic malnutrition. Additionally, dietitians routinely screen admission lists of high‐risk patient groups and generate self‐referrals. Dietitians use an internal triage tool to prioritise the urgency of responding to referrals (unpublished).

### 2.1 Malnutrition Point Prevalence Survey (PPS)

The PPS was conducted on a single day in July 2022 across three campuses of one tertiary health service, Austin Health, Melbourne, Australia. Eligibility criteria for the PPS included ≥ 18 years of age, free from cognitive impairment and fluent in English; cognitive impairment was identified by the PPS dietitians in consultation with the nurse unit manager and review of clinical notes. Patients with an expected length of stay ≤ 2 nights, receiving end‐of‐life care, unable to provide consent or be assessed, in ICU, emergency, short stay, day surgery and spinal units were excluded.

On the day of the PPS dietitians screened patient admission lists for eligible patients and collected data on sex, age, date of admission, ward, unit, campus, date and score of most recent Malnutrition Screening Tool (MST) conducted by nurses [11], dietitian assessment and provided medical nutrition therapy if appropriate from the electronic medical record (EMR). Dietitians obtained verbal consent from eligible patients and completed the MST. For any patients identified at risk of malnutrition via the dietitian completed MST, the dietitian then used validated tools to diagnose malnutrition such as the Subjective Global Assessment (SGA) [20] or the Global Leadership Initiative on Malnutrition (GLIM) [21] when a physical assessment was not able to be completed. Body mass index (BMI) was also calculated by dietitians on the day of the PPS.

### 2.2 Retrospective Case Series

For the retrospective case series, data were collected from the EMR between March and August 2024 by student dietitians [SK and OD] under supervision of a senior dietitian [JB]. Inclusion criteria for this subset analysis were restricted to inpatients diagnosed with malnutrition or deemed at risk of malnutrition by dietitians on the day of PPS that were not receiving dietetic care. Additional data collected for this case series included date and time of initial and subsequent completion of MST by nursing staff, as required by the hospital guideline, dates of dietitian referrals (including cases where a referral was placed but the patient was not seen by the dietitian), dates of recorded weights, and documentation of nutrition impact symptoms such as poor dietary intake. Additional information that was collected from the EMR included clinical stream and length of stay.

*Accuracy of MST responses:* All MSTs completed by nurses between admission and the PPS day were assessed for accuracy by a dietitian based on available weight history and information in the EMR compared with nursing response to the MST, for example, loss of weight evident from previous weights or weight loss or nutrition impact symptoms indicating poor dietary intake documented in medical notes.

*Influence of nursing shift on accuracy of MST:* The time of MST completion and accuracy of all the MSTs completed from admission to discharge were categorised into morning shift (0600–1400 h), afternoon shift (1400–2200 h) and evening shift (2200–0600 h) to assess whether nursing shifts influenced the accuracy of the MST.

### 2.3 Statistical Analysis

All de‐identified data was collected and analysed using Microsoft Excel. No formal power calculation was performed as this was a retrospective audit aimed at describing a real‐world inpatient scenario rather than testing a specific hypothesis. Quantitative descriptive data were presented as proportions, means and standard deviations for parametric data or medians and interquartile ranges for non‐parametric data.

## 3 Results

**Figure 1:**
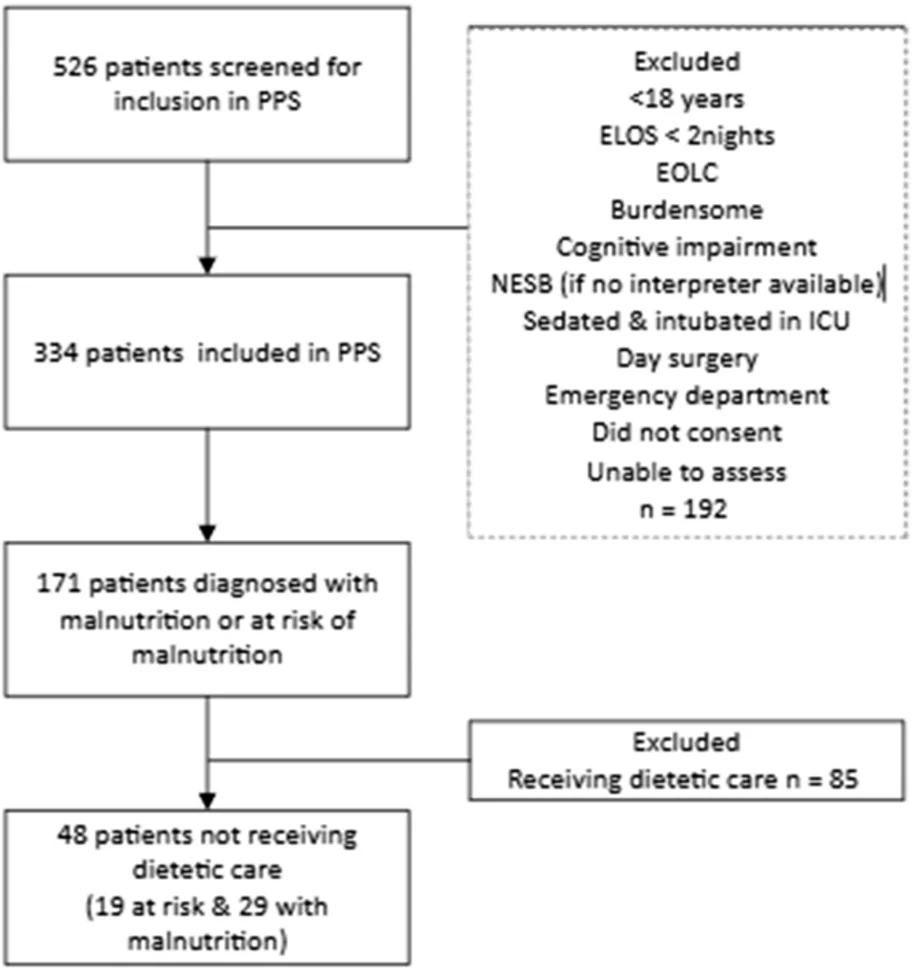
Participant flow diagram.

Of the 171 patients included in the initial PPS audit, a total of 48 patients (28%) were identified as at risk of or with malnutrition and not receiving dietetic care and were included in the retrospective case series analysis, after excluding those with inadequate data (Figure 1). The characteristics of included patients are outlined in Table 1. Of the 48 patients, 54% were male, 73% of patients were located at the acute campus, followed by the subacute campuses (27%). Fifty percent of patients (*n* = 24) were admitted under the medical stream, with similar numbers admitted under subacute (*n* = 12) and surgical stream (*n* = 11). All patients under the cancer stream on the day of PPS with malnutrition or at risk of malnutrition were receiving dietetic care. The median number of days in hospital on the day of the PPS was 5.0 (IQR 2.0, 11.5) days. The median length of stay was 16.5 (IQR 7.8, 28.3) days. Sixty percent of participants (*n* = 29) stayed for > 2 weeks, 23% (*n* = 11) stayed for < 1 week and ~15% (*n* = 7) between 1 and 2 weeks. Fifty‐six percent (*n* = 27) of patients were mildly to moderately malnourished, and 4% (*n* = 2) were severely malnourished. The remaining patients (*n* = 19, 40%) were well‐nourished but at risk of malnutrition.

**Table 1:** Characteristics of the 48 patients with malnutrition or at risk of malnutrition not receiving dietetic care.

Characteristic	Patients (*n* = 48)
Median age years (IQR)	74.5 (62.8, 86.3)
Sex: *n* (%)	
Male	26 (54)
Female	22 (46)
Campus: *n* (%)	
Acute	35 (73)
Subacute	12 (25)
Specialist rehab	1 (2)
Clinical stream *n* (%)	
Medical	24 (50)
Surgical	11 (23)
Cancer	0 (0)
Subacute	13 (27)
Median length of stay days (IQR)	16.5 (7.8, 28.3)
Median days in hospital on day of PPS (IQR)	5.0 (2.0, 11.5)
Malnutrition severity based on SGA, *n* (%)	
Well‐nourished, at risk	19 (40)
Mild/moderate	27 (56)
Severe	2 (4)
Median BMI kg/m^2^ (IQR)	24.7 (21.7, 29.0)

Abbreviations: BMI, body mass index kg/m^2^; IQR, interquartile range; n, number; SGA, Subjective global assessment; %, percentage.

Table 2 depicts the reasons patients at risk of malnutrition or malnourished were not receiving dietetic care on the day of the PPS, based on the accuracy of the MSTs completed prior to the PPS day. Sixty percent (*n* = 29) of patients had not been referred to dietetics due to inaccurate completion of the MST, and one additional patient had not had an MST completed. The main reasons that the MST was deemed inaccurate were that on the day of the PPS the patient or family reported unintentional weight loss that indicated weight loss not obviously related to fluid status, or weight loss was documented in the medical notes (*n* = 17) and/or previous weights were available in the EMR that indicated weight loss (*n* = 13), but these weight changes had not been identified on the MST.

**Table 2:** Reasons the 48 patients at risk of malnutrition or malnourished were not receiving dietetic care on the day of the PPS according to the accuracy of the MSTs completed prior to the PPS day.

Reasons	MST inaccurate *n* (%)	MST accurate *n* (%)	MST not completed *n* (%)
Nursing accuracy of MST at time of PPS	29 (60)	18 (38)	1 (2)
Weights available in EMR contrary to MST response	13 (27)		
Patient/carer reported weight loss on day of PPS or weight loss evident in medical note	17 (35)		
MST not sensitive to chronic malnutrition	1 (2)	6 (13)	
Poor oraI intake without weight loss		4 (8)	
Potential misdiagnosis of malnutrition or malnutrition risk by PPS dietitian		3 (6)	
Referral received day prior to PPS but not yet assessed by dietitian		3 (6)	
Intentional weight loss		1 (2)	
Fluid shifts masking weight loss		1 (2)	

*Note:* some patients had multiple reasons that they were not known to dietetics.

Abbreviations: EMR, electronic medical record; MST, malnutrition screening tool; PPS, point prevalence survey.

Six patients (13%) who had accurate MST(s) completed were not referred to dietetics as the MST questions are not sensitive to chronic malnutrition, for example, one patient had a BMI of 17, and weighed 45 kg. A further four patients also with accurate MSTs were not referred to dietetics as the scoring doesn't trigger a referral when oral intake is poor, but weight loss has not yet been recorded. Three patients whose MST was deemed to be accurate may have had a misdiagnosis of malnutrition or malnutrition risk by the PPS dietitian. In one case, a patient who was diagnosed as SGA‐B (moderate) by the PPS dietitian was assessed as SGA‐ A by the unit dietitian 1 week later; in another, a patient who was SGA‐A at risk had no clear documentation explaining this classification and in the third case, a patient classified as SGA B (mild) had no documented weight loss and nursing notes indicated consistently good oral intake. A further three patients had accurate MSTs and had been referred the day prior to the PPS. These patients had been triaged but not yet assessed by the dietitian, in accordance with the hospital's priortisation tool and its specified review timeframe.

There was variability in time from admission to the first recorded weight (Table 3). Thirty‐five percent (*n* = 17) of patients had a recorded weight within the first 24 h of admission as per hospital guidelines. Among patients with a LOS greater than 1 week, 17% (*n* = 8) were not weighed within their first week of admission, and 4% (*n* = 2) were never weighed during their admission.

**Table 3:** Time from admission to first recorded weight.

First weight recorded	*n* (%)
≤ 24 h	17 (35)
24–48 h	10 (21)
2–7 days	12 (25)
≥ 1 week	6 (13)
Nil weight & LOS ≤ 1 week	1 (2)
Nil weight & LOS > 1 week	2 (4)

Abbreviation: LOS, length of stay.

Table 4 shows the accuracy of all 94 MSTs completed for the 48 patients from admission to discharge by nursing shift. Fifty‐one percent (*n* = 49) of all MSTs were inaccurate. The morning shift had the greatest proportion of inaccurate MSTs (65%), followed by the afternoon shift (48%), and the night shift (36%).

**Table 4:** Accuracy of all MSTs completed for PPS patients from admission to discharge by nursing shift.

Nursing shift	Total	Accurate MSTs *n* (%)	Inaccurate MSTs *n* (%)
AM	34 (36)	12 (35)	22 (65)
PM	46 (49)	24 (52)	22 (48)
Night	14 (15)	9 (64)	5 (36)
Total	94 (100)	46 (49)	48 (51)

Abbreviations: AM, morning shift; night, overnight shift; PM, afternoon shift.

## 4 Discussion

Malnutrition is highly prevalent in hospitalised patients and contributes to significant health and economic burden. Early identification and intervention are recommended as a strategy to mitigate associated complications of malnutrition [22]. This retrospective audit aimed to investigate factors contributing to the under‐identification of patients at risk of or with existing malnutrition and not receiving dietetic care. Within this cohort of patients, 60% indeed had mild‐severe malnutrition. The standard MST screening tool used within the hospital was inaccurately completed for 29 patients, and not completed for an additional patient, meaning 62.5% of MSTs were inaccurate. Key contributors to inaccurate malnutrition screening included instances of reported or documented weight loss, as well as historical weight data available in the EMR indicating weight loss that had not been captured in the MST. These findings highlight broader system‐level challenges, including limited available weight data, time constraints and competing clinical priorities during shifts. Furthermore, only 35% of patients had their weight documented within the first 24 h of admission, despite hospital guidelines requiring completion of the MST within the first 8 h. Across nursing shifts, MST screenings were most frequently inaccurate during the morning shift, potentially reflecting challenges related to nursing workload or capacity at that time.

Of particular concern within this cohort of missed dietetic referral opportunities, two patients were severely malnourished and 27 had mild to moderate malnutrition. A significant contributing factor was the underutilisation of available weight change data, whether reported by patients or family members, or documented in the EMR. This suggests potential issues with the accessibility and visibility of weight history within the EMR. Enhancing staff awareness of where to locate this information may help address this gap in screening accuracy. While all patients who are malnourished or at risk should ideally receive dietetic care, similar gaps have been reported in other studies. For example, an audit of 5186 patients found a 30% shortfall in identifying malnourished individuals, largely attributed to poor completion of screening tools [22]. It has been identified that to complete the MST a large proportion of information is required in addition to information the patient provides, which is especially challenging in the setting of cognitive impairment [23], and highlights the need for ready access to weight history within the EMR.

An additional challenge relates to the sequencing and feasibility of required admission documentation as outlined in existing hospital guidelines. Nursing staff are required to complete a substantial number of assessments upon patient admission, often while managing competing clinical priorities across multiple patients. Evidence suggests that, in such contexts, nutrition‐related tasks may be deprioritised, not due to individual oversight, but due to several barriers including systemic workload pressures and time constraints [24]. The MST is one such assessment required within the first 8 h of admission, whereas initial weight measurement, a critical component of MST accuracy, is only mandated within the first 24 h. This misalignment in task timing introduces a structural barrier to accurate screening. Findings from this audit support this concern, with 35% of patients not weighed within the recommended 24‐h timeframe, and notably, 17% had no recorded weight within the first week of admission. These gaps likely compromise the accuracy of MST completion and subsequent identification of patients at nutritional risk. Rather than increase expectations of nursing staff by requiring weight to be obtained earlier, consideration should be given to adjusting the timing of MST completion to better align with routine clinical workflows. Current research indicates that nutrition screening tools should be completed within 24–48 h of admission [25]; however, given the current 8‐h policy, audit findings suggest screening may be performed inaccurately when undertaken prematurely without sufficient data. Improving malnutrition screening requires a systems‐based approach that prioritises feasibility and accuracy. Adjusting MST timing does not diminish its importance but instead supports a balance between timely and accurate screening. Given that the primary purpose of screening is to enable appropriate dietetic referral, ensuring high‐quality, reliable responses is critical. Aligning MST completion with the availability of accurate weight data and with realistic nursing capacity, may enhance both compliance and accuracy.

Other challenges identified within this audit to enable appropriate dietetic referrals included the impact of nursing capacity. The morning shift performed the worst for MST accuracy potentially reflecting challenges related to nursing workload or capacity at that time of day. A study from the USA indicated that nursing work was not distributed equally across the day, with greater frequency and duration of hands‐on tasks between 7 a.m.–11 a.m. [26]. This pattern may contribute to reduced capacity for accurate completion of admission screening tools during this peak period. These findings present an opportunity to further align screening practices with clinical workflow demands. For example, if there is high demand for admission screening during morning shifts, then protocols may be adjusted to allow this screening to be conducted in the afternoon or night shifts to enhance completion rates and accuracy. As previously suggested, extending the MST completion window from within 8 h to within 24 h of admission would support flexibility, enabling screening to be undertaken at a time when accuracy is more achievable. This adjustment reflects a broader systems‐based approach, prioritising feasible and high‐quality assessment over rigid timing requirements.

Other challenges exist including the sensitivity of the MST tool, whereby the MST questions are not sensitive to chronic malnutrition, and the scoring doesn't trigger a referral when oral intake is poor, but weight loss has not yet been recorded. The sensitivity of the MST has been suggested to be 64% when compared to the GLIM criteria [27], and 60% in another study comparing to the International Classification of Diseases Criteria (ICD‐10‐AM) [23]. Additionally, there were some instances where the MST was accurate, but the dietitians had misdiagnosed malnutrition, suggesting that dietitians may over‐diagnose malnutrition in some cases. This discrepancy may also reflect the variability and potential clinical bias inherent in subjective assessment tools, time constraints or inter‐individual variability in assessments. While the SGA is used globally and is considered by some to be the most validated tool to assess malnutrition in the hospital setting [28], it relies on clinical judgement which can be different depending on training, experience and clinical interpretation, therefore making it a subjective measure [29].

The results of this study should be interpreted within the limitations of the design. First, the sample size is small and from a single centre which may limit the generalisability of the findings. Second, the retrospective nature of the study relies on data available, meaning some data may have been missing or incomplete, potentially impacting the accuracy of the findings. Further to this, the MST and SGA both carry limitations, with the MST influenced by questioning and training, and the SGA being inherently subjective. We also assumed that dietitian‐completed MST was accurate, although the chances of inaccuracy were low given the dietititans had more available weight data. Additionally, overnutrition and associated risk of inadequate intake during admission was not addressed within this cohort [30]. Finally, there is potential for selection bias, as patients with cognitive impairment were not represented in this population group despite attempts to involve family members. Despite these limitations, the study has several strengths, including its integration within a larger point‐prevalence survey of hospitalised patients and the use of validated tools for malnutrition screening (MST) and assessment (SGA). The critical focus within this study on patients who should have received dietetic care but were missed, is a major strength. These features enhance the study's relevance and provide valuable insights into hospital‐wide systems challenges and potential areas for improvement.

The results from this study can inform future clinical quality improvement as well as future research directions. Optimisation of EMR systems should be considered for those hospitals currently using an EMR, as well as for health services yet to implement electronic records. For example, ensuring that weight history is easily accessible and visible to all staff will likely improve nursing staff ability to see changes in weight over time, improving MST accuracy. The current Austin Health EMR system requires nursing staff to re‐enter the entire risk screening bundle (delirium, falls, pressure injury, aggression, infection, mental state, nutrition) when updating a single item, which likely discourages timely revisions. Modifying the system to allow single‐item updates that trigger prompts for related fields may improve usability and support more accurate documentation. Furthermore, improved timing across hospital protocols is required, for example, a swallow assessment prior to the first meal is important to prevent aspiration, but the MST screening tool may be better placed to be completed within 24‐h of admission to align with the requirement for weighing the patient. This is especially relevant given that weight is needed to improve MST accuracy. Future studies should explore system‐based solutions to automatically calculate and flag clinically significant loss of weight from electronic records, and assess whether such alerts are useful, especially given challenges of other clinical reasons for weight loss such as diuresis and amputations. Furthermore, future studies could utilise a qualitative design to comprehensively explore barriers to completing risk screening within healthcare professionals including nursing staff.

## 5 Conclusion

This study has identified several modifiable factors that may improve identification of malnutrition and malnutrition risk as well as subsequent referral rates for dietetic input. Clear gaps exist in the timely and accurate completion of MST screening and weight documentation. A multitude of factors likely influence this including nursing staff capacity, flawed design of EMR systems, and suboptimal protocol sequencing. Immediately translatable recommendations include optimising hospital protocol sequencing and improving visibility of weight records in the EMR. Formal feedback with nursing staff is an important next step to support practice change and should be considered in future quality improvement initiatives. Future research applying broad systems‐level thinking is needed to enhance malnutrition prevention and management, including exploration of how differences in referral patterns across clinical specialties may influence the identification and prioritisation of malnutrition risk.

## Author Contributions

Jacqui Bailey, Caroline J. Tuck, and Katherine J. Desneves designed the study. Jacqui Bailey, Olivia Dullard, Sophie Kane undertook data collection. Jacqui Bailey, Jolie Baird, Olivia Dullard, and Sophie Kane undertook data analysis and interpretation. All authors contributed to analysis and interpretation of the data, drafting and reviewing the manuscript, and approved the final draft of the manuscript submitted for publication. The content prepared within this manuscript has not been published elsewhere.

## Funding

The authors have nothing to report.

## Conflicts of Interest

The authors declare no conflicts of interest.

## Data Availability

The data that support the findings of this study are available on request from the corresponding author. The data are not publicly available due to privacy or ethical restrictions.

## References

[1] World Health Organisation. ‘Malnutrition’ 2022 Available from: https://www.who.int/health-topics/malnutrition [Accessed November 23 2024].

[2] Y. Sharma , M. Miller , R. Shahi , P. Hakendorf , C. Horwood , and C. Thompson , “Malnutrition Screening in Acutely Unwell Elderly Inpatients,” British Journal of Nursing 25, no. 18 (2016): 1006–1014. 27734728 10.12968/bjon.2016.25.18.1006

[3] K. E. Charlton , C. Nichols , S. Bowden , et al., “Older Rehabilitation Patients Are at High Risk of Malnutrition: Evidence From a Large Australian Database,” Journal of Nutrition, Health and Aging 14, no. 8 (2010): 622–628. 10.1007/s12603-010-0307-3 PMC12880241 20922337

[4] S. Marshall , A. Young , J. Bauer , and E. Isenring , “Malnutrition in Geriatric Rehabilitation: Prevalence, Patient Outcomes, and Criterion Validity of the Scored Patient‐Generated Subjective Global Assessment and the Mini Nutritional Assessment,” Journal of the Academy of Nutrition and Dietetics 116, no. 5 (2016): 785–794. 26212447 10.1016/j.jand.2015.06.013

[5] M.‐C. O'Shea , J. Bauer , C. Barrett , et al., “Malnutrition Prevalence in Australian Residential Aged Care Facilities: A Cross‐Sectional Study,” Healthcare 12, no. 13 (2024): 1296. 38998831 10.3390/healthcare12131296 PMC11241761

[6] Royal Commission into Aged Care Quality and Safety . Final Report—Executive Summary Barton, ACT: Australian Government; 2021. https://agedcare.royalcommission.gov.au/sites/default/files/2021-03/finalreport-executive-summary.pdf.

[7] K. Thiele Nutrition for Older Australians—Priorities for the 2022 Federal Budget, https://treasury.gov.au/sites/default/files/2022-03/258735_nutrition_for_older_australians_alliance.pdf.

[8] P. Schuetz , D. Seres , D. N. Lobo , F. Gomes , N. Kaegi‐Braun , and Z. Stanga , “Management of Disease‐Related Malnutrition for Patients Being Treated in Hospital,” Lancet (London, England) 398, no. 10314 (2021): 1927–1938. 34656286 10.1016/S0140-6736(21)01451-3

[9] A. R. Cass and K. E. Charlton , “Prevalence of Hospital‐Acquired Malnutrition and Modifiable Determinants of Nutritional Deterioration During Inpatient Admissions: A Systematic Review of the Evidence,” Journal of Human Nutrition & Dietetics 35, no. 6 (2022): 1043–1058. 35377487 10.1111/jhn.13009 PMC9790482

[10] Australian Commission on Safety and Quality in Health Care. National Safety and Quality Service Standards (2nd ed.). 2021. Available from: https://www.safetyandquality.gov.au/national-standards/nsqhs-standards. [Accessed November 23 2024].

[11] M. Ferguson , S. Capra , J. Bauer , and M. Banks , “Development of a Valid and Reliable Malnutrition Screening Tool for Adult Acute Hospital Patients,” Nutrition 15, no. 6 (1999): 458–464. 10378201 10.1016/s0899-9007(99)00084-2

[12] E. Isenring , G. Cross , L. Daniels , E. Kellett , and B. Koczwara , “Validity of the Malnutrition Screening Tool as an Effective Predictor of Nutritional Risk in Oncology Outpatients Receiving Chemotherapy,” Supportive Care in Cancer 14, no. 11 (2006): 1152–1156. 16622648 10.1007/s00520-006-0070-5

[13] S. Marshall , A. Young , and E. Isenring , “The Malnutrition Screening Tool in Geriatric Rehabilitation: A Comparison of Validity When Completed by Health Professionals With and Without Malnutrition Screening Training Has Implications for Practice,” Journal of the Academy of Nutrition and Dietetics 118, no. 1 (2018): 118–124. 28476323 10.1016/j.jand.2017.03.019

[14] D. S. Peiris , M. J. Wakeling , M. M. Jones , et al., “Evaluation of Accuracy of Nutritional Assessments Using Malnutrition Universal Screening Tool (MUST) Upon Admission to the Parenteral Nutrition Trained Wards in One of the Home Parenteral Nutrition Centers,” Clinical Nutrition ESPEN 61 (2024): 507.

[15] M. Mellor and S. Tanner , “2794 A MUST to Improve Patient Outcomes; A Multidisciplinary Approach to Improving Nutrition,” Age and Ageing 54, no. Suppl_1 (2025): afae277.012.

[16] K. Ylli , C. Sheehan , and T. Nugent , “WP8.3—“Effectiveness of Malnutrition Screen and Interventions for Surgical Inpatients‐A MUST Score Based Audit,” BJS 111, no. Suppl_8 (2024): znae197.214.

[17] M. Frank , A. Sivagnanaratnam , and J. Bernstein , “Nutritional Assessment in Elderly Care: A Must!,” BMJ Quality Improvement Reports 4, no. 1 (2015): u204810.w2031. 10.1136/bmjquality.u204810.w2031 PMC4645872 26734346

[18] H. Arifin , R. Chen , C. M. Sung , K. J. Chiang , and K. R. Chou , “Performance of Malnutrition Screening Tools on People With Chronic Diseases: A Bivariate Meta‐Analysis,” Journal of Nursing Research 34, no. 1 (2026): e440. 10.1097/jnr.0000000000000728 PMC12863617 41627084

[19] E. von Elm , D. G. Altman , M. Egger , S. J. Pocock , P. C. Gøtzsche , and J. P. Vandenbroucke , “The Strengthening the Reporting of Observational Studies in Epidemiology (STROBE) Statement: Guidelines for Reporting Observational Studies,” PLoS Medicine 4, no. 10 (2007): e296. 17941714 10.1371/journal.pmed.0040296 PMC2020495

[20] A. S. Detsky , M. Jr , J. P. Baker , et al., “What Is Subjective Global Assessment of Nutritional Status?,” JPEN 11, no. 1 (1987): 8–13. 10.1177/014860718701100108 3820522

[21] T. Cederholm , G. Jensen , M. Correia , et al., “GLIM Criteria for the Diagnosis of Malnutrition–A Consensus Report From the Global Clinical Nutrition Community,” Journal of Cachexia, Sarcopenia and Muscle 10, no. 1 (2019): 207–217. 30920778 10.1002/jcsm.12383 PMC6438340

[22] K. Connell , A. Elliott , E. McShane , A. Bramley , L. Hanna , and K. Furness , “Screening, Characterising and Assessing Malnutrition in the Hospital Setting: A Large‐Scale Point Prevalence Survey,” Nutrition & Dietetics 83, no. 3 (2026): 326–333. 40855974 10.1111/1747-0080.70045

[23] J. J. Bell , J. D. Bauer , S. Capra , and R. C. Pulle , “Quick and Easy Is Not Without Cost: Implications of Poorly Performing Nutrition Screening Tools in Hip Fracture,” Journal of the American Geriatrics Society 62, no. 2 (2014): 237–243. 24428255 10.1111/jgs.12648

[24] S. M. Green and E. P. James , “Barriers and Facilitators to Undertaking Nutritional Screening of Patients: A Systematic Review,” Journal of Human Nutrition & Dietetics 26, no. 3 (2013): 211–221. 23331465 10.1111/jhn.12011

[25] C. Serón‐Arbeloa , L. Labarta‐Monzón , J. Puzo‐Foncillas , et al., “Malnutrition Screening and Assessment,” Nutrients 14, no. 12 (2022): 2392. 35745121 10.3390/nu14122392 PMC9228435

[26] P.‐Y. Yen , M. Kellye , M. Lopetegui , et al., editors. “Nurses' Time Allocation and Multitasking of Nursing Activities: A Time Motion Study,” in *AMIA Annual Symposium Proceedings*, (2018), 1137–1146. PMC6371290 30815156

[27] C. van Dronkelaar , M. Tieland , T. Cederholm , E. M. Reijnierse , P. J. Weijs , and H. Kruizenga , “Malnutrition Screening Tools Are Not Sensitive Enough to Identify Older Hospital Patients With Malnutrition,” Nutrients 15, no. 24 (2023): 5126. 38140387 10.3390/nu15245126 PMC10745606

[28] J. P. Allard , H. Keller , L. Gramlich , K. N. Jeejeebhoy , M. Laporte , and D. R. Duerksen , “GLIM Criteria Has Fair Sensitivity and Specificity for Diagnosing Malnutrition When Using SGA as Comparator,” Clinical Nutrition ESPEN 39, no. 9 (2020): 2771–2777. 10.1016/j.clnu.2019.12.004 31918864

[29] J. da Silva Fink , P. D. de Mello , and E. D. de Mello , “Subjective Global Assessment of Nutritional Status–A Systematic Review of the Literature,” Clinical Nutrition ESPEN 34, no. 5 (2015): 785–792. 10.1016/j.clnu.2014.12.014 25596153

[30] R. N. Dickerson , L. Andromalos , J. C. Brown , et al., “Obesity and Critical Care Nutrition: Current Practice Gaps and Directions for Future Research,” Critical Care 26, no. 1 (2022): 283. 36127715 10.1186/s13054-022-04148-0 PMC9486775

